# Exploring Endophytic Fungi as Natural Antagonists against Fungal Pathogens of Food Crops

**DOI:** 10.3390/jof10090606

**Published:** 2024-08-26

**Authors:** Kumudu K. Manathunga, Niranjan W. Gunasekara, Muditha K. Meegahakumbura, Pamoda B. Ratnaweera, Turki Kh. Faraj, Dhanushka N. Wanasinghe

**Affiliations:** 1 Department of Science and Technology, Faculty of Applied Sciences, Uva Wellassa University, Badulla 90000, Sri Lanka; kkumudu1996@gmail.com (K.K.M.); pamoda@uwu.ac.lk (P.B.R.); 2 Department of Export Agriculture, Faculty of Animal Science and Export Agriculture, Uva Wellassa University, Badulla 90000, Sri Lanka; niranjan@uwu.ac.lk; 3 Department of Soil Science, College of Food and Agriculture Sciences, King Saud University, P.O. Box 145111, Riyadh 11362, Saudi Arabia; talasiri@ksu.edu.sa; 4 Honghe Center for Mountain Futures, Kunming Institute of Botany, Chinese Academy of Sciences, Honghe 654400, China

**Keywords:** bio-fungicides, crop resilience, environmental health, phytopathogen suppression, plant protection, sustainable agriculture, antagonistic mechanism

## Abstract

The yield and quality of cultivated food crops are frequently compromised by the prevalent threat from fungal pathogens that can cause widespread damage in both the pre-harvest and post-harvest stages. This paper investigates the challenges posed by fungal pathogens to the sustainability and yield of essential food crops, leading to significant economic and food security repercussions. The paper critiques the long-standing reliance on synthetic fungicides, emphasizing the environmental and health concerns arising from their widespread and occasionally inappropriate use. In response, the paper explores the potential of biological control agents, specifically endophytic fungi in advancing sustainable agricultural practices. Through their diverse symbiotic relationships with host plants, these fungi exhibit strong antagonistic capabilities against phytopathogenic fungi by producing various bioactive compounds and promoting plant growth. The review elaborates on the direct and indirect mechanisms of endophytic antagonism, such as antibiosis, mycoparasitism, induction of host resistance, and competition for resources, which collectively contribute to inhibiting pathogenic fungal growth. This paper consolidates the crucial role of endophytic fungi, i.e., *Acremonium*, *Alternaria*, *Arthrinium*, *Aspergillus*, *Botryosphaeria*, *Chaetomium*, *Cladosporium*, *Cevidencealdinia*, *Epicoccum*, *Fusarium*, *Gliocladium*, *Muscodor*, *Nigrospora*, *Paecilomyces*, *Penicillium*, *Phomopsis*, *Pichia*, *Pochonia*, *Pythium*, *Ramichloridium*, *Rosellinia*, *Talaromyces*, *Trichoderma*, *Verticillium*, *Wickerhamomyces*, and *Xylaria*, in biological control, supported by the evidence drawn from more than 200 research publications. The paper pays particular attention to *Muscodor*, *Penicillium*, and *Trichoderma* as prominent antagonists. It also emphasizes the need for future genetic-level research to enhance the application of endophytes in biocontrol strategies aiming to highlight the importance of endophytic fungi in facilitating the transition towards more sustainable and environmentally friendly agricultural systems.

## 1. Introduction

Food crops play a crucial role in global nutrition and food security, and they are being cultivated in a wide variety to meet the diverse dietary needs of the global population. The United Nations Food and Agriculture Organization (FAO) identifies rice, wheat, maize, and sugarcane as the most commonly grown food crops globally [1]. Additionally, fruits are essential according to the dietary guidelines of several countries, due to their nutritional value, distinctive flavors, and health benefits derived from their antioxidant properties [2]. Similarly, vegetable crops are grown worldwide as they are a vital source of fiber and nutrients, i.e., vitamins, minerals, and antioxidants, highlighting their importance in a balanced diet [3]. While performing the leading role in global food security, food crops play a critical role in global markets and sustaining livelihoods, particularly in agrarian societies [4].

The challenge of food scarcity is made worse by biotic and abiotic stresses, which include plant pathogens, nutrient deficiency or excesses, and limited land and water resources. Environmental sustainability issues and climate change phenomena, such as extreme temperatures, flooding, drought, and salinity also affect food scarcity [5,6]. Among these challenges, plant diseases are particularly detrimental and cause qualitative and quantitative reductions in crop production, with severe economic losses. Fungal diseases play a significant role in this context [7,8,9].

Despite their essential function in the cycling of nutrients within ecosystems, fungi also act as parasites, pathogens, and predators by creating challenges to various ecosystems and ultimately increasing global food insecurity [10]. With over 19,000 species identified as pathogenic fungi, fungi-like organisms are responsible for approximately 70–80% of all plant diseases. This highlights the extensive impact they have on the health and productivity of food crops [11,12]. The severity of fungal infections on food crops is increasing by causing significant barriers to crop yield and quality and to the advancement of sustainable agriculture [12]. Fungal pathogens can infect almost all the plant parts, causing a wide range of disease symptoms, such as wilting, rotting, discoloration, deformation, and the formation of wounds. Some of the most common fungal diseases that affect food crops include leaf blights, anthracnose, powdery mildew, downy mildew, rusts, Rhizoctonia rots, early blights, and Fusarium wilt. These diseases present significant challenges to maintaining crop health and productivity, and they emphasize the critical need for effective disease management strategies to safeguard global food security [11,13].

Due to the severity of the fungal diseases, farmers have historically employed numerous management strategies to control them, including cultural practices and the use of synthetic fungicides [7,8]. Cultural control techniques, such as crop rotation, sanitation, tillage, enhancing crop growth conditions, and selecting resistant cultivars have been foundational in managing fungal diseases [14]. However, the emergence of new fungal diseases and growing concerns over the adverse effects of synthetic fungicides on both the environment and human health have driven a shift towards sustainable agricultural practices. In response to these challenges, biocontrol agents have been increasingly recognized and utilized as effective commercial solutions to mitigate the impact of fungal diseases. This marks a significant advancement in the field of sustainable agriculture [8]. Bio-control methods are significant because of their unique characteristics, such as safety, limited host range or target specificity, the absence of hazardous residues, eco-friendliness, and ease of application [15].

Exploration into new biodiversity niches has led to the discovery of novel bioactive secondary metabolites from endophytes, highlighting the vast and largely unexplored diversity of endophytic fungi [16]. Endophytic fungi live in the internal tissues of plants, establishing a symbiotic relationship without causing any symptoms of disease to the host [17]. However, endophytes may become pathogenic under favorable environmental conditions and weakened plant health [17]. These fungal endophytes engage in mutualistic symbiotic relationships with their host plants by contributing to plant health through directly producing essential phytohormones and enhancing nutrient uptake and photosynthetic efficiency. Indirectly, they play a role in increasing secondary metabolite production and supporting plant defenses against a range of biotic stresses and abiotic stresses, i.e., heavy metal contamination and dehydration [18]. Of the estimated 2.2–3.8 million fungal species on Earth [19], about 1 million are believed to be endophytic and inhabit plants in diverse environments ranging from seagrasses to lichens and palms [17,20,21]. Endophytes are capable of producing secondary metabolites that have shown significant potential in inhibiting some of the most destructive plant pathogens [20,22]. In modern agriculture, these antagonistic fungi are increasingly recognized and used as biocontrol agents due to their potent inhibitory action, especially those with multiple mechanisms for controlling phytopathogens [23,24].

This comprehensive review aims to gather and enhance existing information on the functions of endophytic fungi, particularly their ability to mitigate the impact of phytopathogenic fungi on food crops. It sets out to clarify the complex mechanisms through which endophytes protect food crops from fungal pathogens, including the direct suppression via antifungal metabolites, competition for resources and the induction of plant defense responses. With this main objective, this review is aimed at identifying existing gaps in the literature and suggesting opportunities for future research and experimental work. A significant portion of this review explores the biological interactions and the dynamics within plant ecosystems with the potential applications of endophytic fungi for enhancing crop health and yield. With the above goals, the article discusses about promoting the advancement of more environmentally friendly agricultural practices by reducing reliance on synthetic fungicides. It will expand our understanding of natural biocontrol strategies with the use of endophytic fungi.

## 2. Materials and Methods

The study on the antagonistic activity of endophytic fungi against pathogenic fungal species of food crops involved thorough data collection from various sources. They comprised a diverse range of formats, i.e., book chapters, review articles, research articles, case studies, reports, conference proceedings, abstracts, manuscripts, and research dissertations that were published in English from 1980 to 2023. This included a wide range of studies, such as theoretical, observational, and experimental to provide a better understanding of the antagonistic mechanisms of endophytic fungi against phytopathogens. The quality of the gathered information was assured by using peer-reviewed materials.

Articles were mainly obtained from prominent academic databases, including ScienceDirect, Google Scholar, ResearchGate, and Web of Science, and it comprised a collection of about 200 research publications focused on endophytic fungi, phytopathogens, and their interactions, especially in food crops. A combination of keywords that included ‘endophytic fungi’, ‘phytopathogens’, ‘antagonistic mechanisms’, ‘antibiosis’, ‘*Trichoderma*’, ‘*Fusarium*’, and ‘penicillium-like fungi’ were mainly used to extract information from the studies. Additional keywords related to cereal crops, such as ‘rice’, ‘maize’, and ‘wheat’, and fruits and vegetables, i.e., ‘banana’, ‘apple’, and ‘bean’, were used in different combinations with the previously mentioned keywords to filter the most relevant publications. The appropriate use of Boolean operators including “and” and “or” allowed for the refinement and expansion of the search results. As an example, the “food crops”, “endophytic fungi”, and “pathogenic fungi” combination expanded the exploration to cover research on both beneficial and harmful fungus in association with food crops.

After thoroughly referring to the gathered literature, important content relevant to the topic was extracted and summarized. Microsoft Excel sheets (Version 2016, Microsoft Corporation, Redmond, WA, USA) were utilized to organize important information relevant to criteria, such as endophytic fungi, crop, pathogenic fungi, fungal disease, disease symptoms, antagonistic mechanism, and/or bioactive agents. Moreover, the consistency of the information was ensured by thoroughly reviewing the content by multiple reviewers.

Studies were grouped based on commonalities, including crop types (cereals, fruits, vegetables), fungal species (i.e., *Muscodor*, *Penicillium*, *Trichoderma*), type of fungal interactions (endophytes, pathogens), and antagonistic mechanisms. This grouping method made it easier to analyze the particular relationships and interactions of endophytes. The contextual considerations, including fungal diseases of same type of crops, antagonistic mechanisms used by endophytes, and the inhibition rates were carefully analyzed to elucidate the outcome of this review.

Accordingly, the results of numerous investigations were analyzed to yield a comprehensive insight into the effectiveness, constraints, and future directions of using endophytes as biocontrol agents against pathogenic fungi in food crops. Identifying incongruities can contribute to shaping future studies in this field and advance our understanding of the factors affecting the efficacy of the application of endophytes as biocontrol agents.

## 3. Endophytic Fungi

The definition of endophytes has undergone a series of changes along with research advancements, leading to several disparities [25]. They have been broadly defined as organisms that reside within plant tissues for part of their lifecycle without causing disease, often benefiting their hosts [26,27,28]. These microbes, including fungi, bacteria, and actinomycetes can occupy ecological niches similar to those of phytopathogens, which highlights their potential as biocontrol agents [29,30]. Endophytic fungi, in particular, have been found in virtually every plant studied, colonizing various plant organs such as stems, leaves, fruits, flowers, rachis, xylem vessels, ovules, tubers, roots, barks, and seeds asymptomatically and periodically [17,30]. The oldest record of mutualistic or endophytic association (endotrophy) of fungi in roots of fossil tree *Amyelon radicians* has been traced to the Paleozoic era [31,32].

These fungi establish saprophytic, mutualistic, or commensalistic relationships with their host plants [33]. Remarkably, every one of the over 300,000 recorded plant species on Earth, including those in diverse environments, i.e., oceans, deserts, tropical rainforests, and polar regions, hosts at least one type of endophyte [17,29,34]. The first comprehensive study of endophytic fungi was conducted in 1904 from the seeds of *Lolium temulentum* [35]. Endophytic fungal diversity is notably higher in tropical regions, presenting challenges in establishing general patterns across different geographical areas [24]. Tropical rainforest ecosystems, which harbor over half of all species on Earth, support a rich variety of endophytic species that produce a higher number of secondary metabolites and more potent natural products compared to those in temperate regions [17].

### 3.1. Ecological Role of the Endophytic Fungi

Endophytic fungi provide direct and indirect benefits to their host plants. Enhancing macro- and micronutrient uptake as well as increasing plant photosynthetic activity, siderophore production, and phytohormone production are some of the direct benefits provided by endophytes to their hosts. Improved resistance to biotic and abiotic stresses, enhanced production of secondary metabolites, and the activation of plant active defense mechanisms are some indirect benefits of endophytes [18,29]. Accordingly, endophytic fungi may provide increased growth and yield in their host plants [36]. A few examples of phytohormones synthesized by the endophytic fungi are *Cladosporium sphaerospermum*, which is an endophyte of *Glycine max*, that produces and releases gibberellic acid for stimulating the plant growth of rice and soybean, while *Piriformospora indica* root endophyte increases the absorption and translocation of nutrients such as nitrogen and phosphorus [37]. Table 1 shows some examples of phytohormones that are produced by endophytes with their function.

Endophytes produce a variety of different bioactive compounds which may be required to overcome biotic and/or abiotic stresses [17]. Biotic stresses include herbivores, insects, pests, nematodes, and other bacterial or fungal pathogens. Abiotic stresses include drought, salinity, and heat stresses. Some examples of endophytes controlling the biotic and abiotic stresses of plants can be given as follows: *Fusarium oxysporum* endophytic fungi decrease the nematocidal activity of *Meloidogyne incognita* in tomatoes. The combination of *Trichoderma atroviride* and *Fusarium oxysporum* endophytes increases the crop yield of bananas with the reduction in *Radopholus similis* nematode population. Endophytic *Curvularia* spp. manages the temperature stress of plants, such as watermelons, tomatoes, and wheat [37].

Endophytic fungi also have the potential to produce a rich variety of secondary metabolites, which have different bioactivities with unknown ecological functions [33,34]. These metabolites belong to different chemical classes, such as alkaloids, flavonoids, terpenoids, phenolics, and steroids [34]. Numerous bioactive properties, including antimicrobial, antiparasitic, antioxidant, anticancer, anti-inflammatory, and immunosuppressive properties are shown by endophytic fungi. The advantages of these bioactive metabolites may include low toxicity, eco-friendly, inexpensive, and lower risk of developing microbial resistance compared to the traditional antimicrobial compounds [29,37]. Table 2 lists some examples of the bioactive secondary metabolites that are produced by endophytic fungi in food crops.

### 3.2. Factors Influencing the Effectiveness of Endophytic Fungi in Plant Disease Control

The effectiveness of endophytic fungi as biocontrol agents is influenced by various factors that can vary widely in nature. These factors include colonization patterns and host specificity, which determine how effectively endophytes can establish and maintain their presence within a particular host plant. The ability of these fungi to migrate within host tissue and trigger the systemic resistance of the plant is essential for their protective function against pathogens [29]. The population dynamics of endophytic fungi can be significantly influenced by competition with other microorganisms and the physical structure of plant tissues. Environmental conditions including temperature, humidity, and light can indeed influence the activity and survival of endophytic fungi. Sui et al. [47] investigated the potential transition from mutualism to commensalism in plant–fungal entomopathogen interactions within agroecosystems. They focused on maize and *Beauveria bassiana* endophytic fungi, which showed a mutualistic relationship under the influence of high air temperatures. Their study highlights that species interactions in agroecosystems are particularly influenced by climate change with elevated temperatures by altering the dynamics between fungal endophytes and plants. These results provide valuable insights for developing future agroecosystem management strategies and enhance our understanding of how the relationships between crops and fungal endophytes are changing in response to climate change [47]. Additionally, the growth phase and physiological state of the host plants are critical factors. Plants in different stages of growth or under varying degrees of stress may respond differently to colonization by endophytes. This variability can significantly impact the overall outcome of the symbiotic relationship between host plants and endophytes. This highlights the importance of comprehensive research and theapproaches used in different agricultural settings [29].

## 4. Pathogenic Fungal Species in Food Crops

Fungal pathogens pose a significant threat to a majority of the commonly cultivated food crops worldwide. They cause substantial yield losses, diminish crop quality, and increase the susceptibility to subsequent infections. They can affect any part of a plant, including fruits, leaves, roots, stems, and barks [48].

### 4.1. Leaf Fungal Diseases

When fungi infect leaves, plants experience a significant reduction in internal activities, which frequently results in harm to the entire plant [11]. The most prevalent fungal diseases affecting plant leaves include anthracnose, blights, rusts, leaf spots, galls, cankers, mildews, coils, and damping-off [11,13,49]. Some of the common fungal diseases affecting plant leaves are briefly described as follows:

**Anthracnose**: Fungi belonging to the genus *Colletotrichum* are the causative agent of the anthracnose disease [50,51]. Anthracnose disease-infected leaves exhibit dark-colored spots, accompanied by browning, curling, and premature dropping. Additionally, infected tissues may experience drying and wilting [52]. It affects a variety of food crops, including beans, tomatoes, lettuce, cucurbits, coffee, avocados, mangoes, strawberries, bananas, guavas, papayas, and capsicums [53,54,55,56,57,58]. Anthracnose can significantly reduce the quality and marketability of the affected crops by leading to considerable yield losses. For example, anthracnose disease in mangoes has been reported to cause up to 100% yield losses [59], while yield losses in chili peppers can range from 10 to 80% [60]. Similarly, avocados can suffer yield losses of up to 60% due to anthracnose [61], and beans can experience 100% yield losses [62]. The *Colletotrichum* species is considered one of the top ten phytopathogens capable of infecting nearly 3000 plant species, resulting in substantial yield reductions in food crops [63].

**Powdery mildew**: This disease is caused by fungal pathogens belonging to various genera, including *Erysiphe*, *Leveillula*, *Microsphaera*, *Sphaerotheca*, and *Oidium* [64,65]. Upon infection with this disease, a white-to-grey powdery coating is developed, typically by covering the upper surface of the infected leaves. Additionally, small and tightly clustered flower buds may develop a white coating of mildew by preventing their opening. Plants with severely infected leaves become dark when they eventually fall. Mature leaves are often unaffected by powdery mildew disease. It prefers to infect young plant tissues that are moist. This fungal disease affects many food crops, such as peas, beans, grapes, strawberries, blackcurrants, apples, and cucumbers [11,13]. *Blumeria graminis* f. sp. *tritici* causes powdery mildew disease in wheat, which can result in up to 40% yield losses in wheat-producing regions globally [66]. Additionally, *Erysiphe pisi*, *E. trifolii*, and *E. baeumleri* species cause this disease in peas, resulting in 25–50% yield losses [67].

**Leaf rust**: Several well-known fungal genera, such as *Cronartium*, *Gymnosporangium*, *Hemileia*, *Puccinia*, and *Uromyces* are responsible for causing rust diseases [68,69]. Symptoms initially appear as slightly raised spots on leaves. Easily identifiable rust-colored lesions are the most significant symptom of this disease. If left untreated, the fungal infection can lead to severe yield losses due to the damage caused to the plant leaves. Leaf rust disease spreads rapidly, particularly in high humidity and moderate temperature conditions. A variety of food crops, including wheat, barley, beans, carrots, and coffee, are susceptible to this disease [11,54]. Recent research studies indicate that rust disease has a significant economic impact on the productivity of food crops. For example, *Puccinia triticina* is responsible for significant production losses in wheat globally, with estimated losses ranging from 10 to 30% [70]. *Hemileia vastatrix* has caused severe damage to coffee, leading to yield losses of up to 80% [71]. Managing leaf rust disease has become challenging due to the continuous evolution of new pathogenic strains with the potential to overcome resistance genes in host plants [72].

**Leaf spots**: Numerous genera of fungi, including *Actinopelte*, *Alternaria*, *Ascochyta*, *Cercospora*, *Cercosporella*, *Cladosporium*, *Corynespora*, *Cylindrocladium*, *Cylindrosporium*, *Didymella*, *Epicoccum*, *Macrophoma*, *Marssonina*, *Phyllosticta*, *Pleospora*, *Ramularia*, and *Septoria*, are responsible for causing leaf spots in plants [73]. Generally, leaf spots become visible approximately a week after the first arrival of the disease. They initially appear as white to greyish white patches on borders. Those spots are surrounded by reddish, brown, or yellowish borders [11]. Fungal leaf spot disease affects various food crops such as peanut, apple, mango, capsicum, celery, gooseberry, tomato, muskmelon, radish, cabbage, bean, peanut, and soybean [11,73]. For example, early and late leaf spots in groundnuts are caused by *Cercospora arachidicola* and *C. personatum*, respectively, which are responsible for yield reductions in groundnuts by up to 70% [74]. Similarly, cercospora leaf spot disease caused by *Cercospora canescens* can result in yield losses of up to 70% in mung beans [75]. Additionally, ramularia leaf spot disease in barley is characterized by a reduction in grain size and increase in yield losses, which can peak at 75% under certain climatic conditions [76].

### 4.2. Root Fungal Diseases

Similar to leaf fungal diseases, fungal root infections also pose significant threats to food crops. They can cause substantial yield and economic losses. Some of the common fungal diseases affecting plant roots are briefly described as follows:

**Fusarium wilt disease**: This is caused by the soil-borne fungus *Fusarium oxysporum*, which is regarded as one of the most harmful fungal root diseases, and it can be considered the most common vascular wilt disease in plants [11,54,77]. Over 100 host-specific strains of *Fusarium oxysporum* are distributed globally [78]. It can develop in warm weather at around 28 °C and can affect the plant at any stage of its growth. The primary symptom of this disease is the internal discoloration of plant vascular bundles [79]. The leaves of infected plants lose their turgidity, droop, and become limp, displaying a lighter green to greenish yellow discoloration. Subsequently, they turn yellow, then brown, and ultimately wilt due to the death of the plant [11]. Potatoes, tomatoes, beans, bananas, cucumbers, legumes, lettuce, eggplants, chickpeas, oil palms, strawberries, watermelons, and other crops are severely affected by Fusarium wilt disease [11,54,77,79]. *Fusarium oxysporum* ranks fifth among the top ten fungal phytopathogens globally [80]. Recent studies have indicated that Fusarium wilt is a highly destructive fungal disease that can cause extensive yield losses. For instance, the yield loss of lentil plants can reach up to 50–100% due to the infection of Fusarium wilt disease [81]. *Fusarium oxysporum* f. sp. *niveum* pathogen is responsible for causing 30–80% yield losses in watermelons [80].

**Rhizoctonia root rot disease**: This is another prominent fungal root disease caused by the *Rhizoctonia solani* fungal pathogen. These fungi attack plant roots by causing them to rot and making it difficult for the plants to absorb water and nutrients. This often leads to wilting and eventual death of the infected plants [82,83]. Its symptoms cannot be observed in some plants until harvesting, because most of them occur below the soil surface [84]. However, stalk discoloration can be observed in sugar beets due to this disease [83]. Rhizoctonia root rot disease damages various food crops, including soybean, rice, potato, apple, tomato, pea, sugar beet, canola, barley, pepper, okra, coriander, chili, fennel, cabbage, and wheat [84,85,86]. This is a devastating disease which can significantly reduce crop yields. For example, up to 60% yield losses can occur in sugar beets [83], while potatoes and rice can experience yield losses ranging from 15 to 50% [84].

**Phytophthora root rot**: This is an aggressive fungal disease of the roots caused by the soil-borne pathogen *Phytophthora* spp. Wilting, branch dieback, and yellow foliage are common symptoms of this disease [87]. It leads to root decay, discoloration of cambiums, stunting, wilting, chlorosis and browning of needles [88]. For instance, infection with phytophthora root rot disease can cause seedling damping-off, seed decay, brown stem lesions, leaf yellowing, root rot, wilting, and eventual plant death in soybean plants [89]. It also causes brown rot in fruits, root rot, and gummosis in citrus during infection [87]. A wide range of food crops, such as avocado, strawberry, citrus, potato, lima bean, string bean, soybean, pepper, pineapple, apple, and cocoa are susceptible to phytophthora root rot disease [73,87,89,90]. This disease causes extensive crop losses, estimated at up to 90%, resulting in an annual loss of US$100 million for susceptible crops [91].

### 4.3. Stem Fungal Diseases

Except for leaf and root fungal diseases, some fungi also attack the stems of plants. Some of the common stem fungal diseases are briefly described below.

**Stem canker disease**: In soybeans, stem canker disease is a critical fungal disease caused by two subspecies of *Diaporthe phaseolorum* [92]. Dark brown, sunken cankers appear on the lower nodes of soybeans as symptoms of stem canker disease. In northern stem canker disease, the cankers are elongated and girdle the stem. Additionally, necrosis and interveinal chlorosis are observed in this disease [93].

**Stem rust disease**: Stem rust, also known as black rust, is a devastating fungal stem disease in wheat and small grain cereal crops. It can decrease grain yield as well as root and foliage growth in wheat plants [94,95]. *Puccinia graminis* f. sp. *tritici* is the causative agent of stem rust disease in wheat. This disease is characterized by elongated, blister-like pustules or uredinia that appear on leaf sheaths, stems, awns, glumes, and leaves of the wheat plant [94].

**Stem rot disease:** The fungal pathogen *Sclerotinia sclerotiorum* causes stem rot disease in food crops, including potatoes, tomatoes, soybeans, beans, lettuce, broccoli, cucurbits, and cabbages. It results in wet rot on stems, fruits, flowers, and leaves [92]. Further, the fungal species *Neocosmospora rubicola* causes stem rot disease in potatoes, resulting in up to a 20% yield loss during production [96].

### 4.4. Fruit Fungal Diseases

A higher intake of fruits and vegetables is recommended as an essential component of a healthy diet because diets rich in these foods are associated with a lower risk of chronic diseases [97]. Globally, approximately 14% of food is wasted from harvest to sale [2]. Fruits with high sugar and nutritional content, along with low pH values, are particularly susceptible to fungi that induce spoilage [98]. Annually, an estimated 25% of fruits are lost due to fungal infections during both the production and postharvest stages [2]. Fungal trunk diseases (FTD) pose an increasing threat to the cultivation of various crops, including berry, citrus, grapevine, nut, olive, pome fruits, and stone fruits. Some of these diseases are summarized in Table 3. Various fungal families, including *Botryosphaeriaceae*, *Calosphaeriaceae*, *Diaporthaceae*, *Diatrypaceae*, *Nectriaceae*, *Phaeomoniellaceae*, *Pleosporaceae*, *Togniniaceae* and *Valsaceae* contaminate host wood in FTD. These fungi primarily infect wounds and later colonize woody tissues, leading to symptoms such as blights, cankers, diebacks, gummosis, and wood rotting [99]. Additionally, blue mold disease affects various fruit species, and it is caused by numerous fungal pathogens. Among them, *Penicillium expansum* is known to affect apples, kiwi, and stone fruits, while *P. italicum* primarily infects citrus fruits. Furthermore, *Penicillium digitatum* is responsible for causing green mold disease in citrus fruits [2]. *Venturia inaequalis* is a global pathogen, causing apple scab disease in apples. It can result in the loss of up to 70% of the total fruit value. Wilt disease in banana plants is a critical fungal infection caused by *Fusarium oxysporum* f. sp. *cubense*. During the 2007–2009 period, it led to a reduction of more than 60% in global banana production [100]. Numerous fungi, including *Agrostalagmus*, *Alternaria*, *Aspergillus*, *Cladosporium*, *Colletotrichum*, *Cylindrocarpon*, *Diaporthe*, *Dothiorella*, *Geotrichum*, *Geotrichum*, *Lasiodiplodia*, *Monilia*, *Mucor*, *Neurospora*, *Peronophythora*, *Stemphylium*, and *Trichoderma*, cause infections on lychee fruits [2]. The fungal species *Phytophthora palmivora* is responsible for black pod disease, which is a devastating fungal disease in cocoa. It can lead to a 44% reduction in global production of cocoa [100,101]. Similarly, stem rot disease in vanilla caused by *Fusarium oxysporum* f. sp. *vanilae* has become a great obstacle for vanilla cultivation, leading to yield losses [100]. Moreover, the *Cholletotrichum* species is responsible for causing anthracnose disease in a variety of tropical fruit crops, including avocados, bananas, mangoes, guavas, papayas, and dragon fruits, as well as temperate fruit crops such as apples, strawberries, cranberries, peaches, and grapes [57,102].

### 4.5. Fungal Diseases in Vegetable Crops

Most vegetables are susceptible to fungi-related diseases, including anthracnose, botrytis rots, downy mildew, Fusarium rots, powdery mildew, rusts, Rhizoctonia rots, sclerotinia rots, and sclerotium rots [54]. Table 3 includes the common fungal diseases that affect certain vegetable crops. White leaf spot disease, which is caused by the *Cercospora*, *Cercosporella*, or *Neopseudocercosporella* species affects cruciferous vegetables, and it is more severe on mustard and turnip. Symptoms of this disease, include small, circular, dry, and pale grey to straw-colored spots. Severely affected leaves turn yellow, dry out, and wither, while still attached to the plant [73,103]. Fusarium rots and wilts caused by the *Fusarium* species are known to affect beans, carrots, cucurbits, onions, tomatoes, and potatoes [79,104]. Additionally, damping-off caused by *Aphanomyces*, *Fusarium*, *Pythium*, *Phytophthora*, or *Rhizoctonia* is prevalent in various vegetable crops, including bean, beetroot, carrot, coriander, eggplant, tomato, spring onion, and leafy vegetables [54,73]. The ascomycete fungus *Sclerotinia sclerotiorum* is capable of infecting up to 408 plant species, including several important crops, such as rapeseed, sunflower, and soybean, along with a wide variety of vegetables. This fungus causes stem rot in the stems of infected plants or water-soaked lesions on their leaves. The most prominent symptoms of *Sclerotinia sclerotiorum* disease in plants are necrotic tissues covered with areas of fluffy white mycelium [105]. Anthracnose disease caused by *Cholletotrichum* species can result in considerable yield losses of up to 100% in beans and 10–80% in chili peppers [62,106].

### 4.6. Fungal Diseases in Cereal Crops

Due to fungal infections, a 15–20% estimated yield reduction may be caused in cereal crops, which can reach up to 60% in extreme cases [107]. Fungal infections in cereal crops represent a significant concern to global food security by reducing grain yield and quality, as well as contaminating human and animal food products. As shown in Table 3, some common fungal diseases that affect cereal crops, include Fusarium head blight (FHB), stem rust, strip rust, powdery mildew, and septoria leaf spot. FHB also known as scab, is caused by the fungus *Fusarium graminearum*. It affects wheat, barley, and other minor grains by causing considerable yield losses, grain quality degradation, and mycotoxin contamination. Symptoms of FHB include necrotic lesions and bleached heads in wheat [108,109].

Wheat and other cereal crops are susceptible to stem rust disease, which is caused by *Puccinia graminis* f. sp. *tritici*. In the Middle East, Africa, and Asia, it has caused significant crop losses and poses a threat to food security. This fungus has developed new, more aggressive strains in recent years that have the potential to spread rapidly [110]. Moreover, both wheat and barley are affected by the fungus *Puccinia striiformis* f. sp. *tritici*, causing strip rust disease. It is particularly common in cooler, wet regions of the world, resulting in severe production losses. Strip rust outbreaks have been reported in many countries, including Australia, Canada, China, and USA [111]. Cereal crops, including oats, wheat, rye, and barley, are susceptible to powdery mildew disease caused by different fungal species including *Blumeria graminis* [112]. Further, septoria leaf spot disease caused by *Septoria tritici* is another fungal disease that seriously affects wheat [113]. Rice blast is another serious disease particularly affecting rice-growing regions, caused by the *Magnaporthe oryzae* (=*Pyricularia oryzae*) fungal species. It results in a reduction of 10–30% in annual global rice production [109,114].

**Table 3:** Fungal diseases of a few economically important food crops with their symptoms.

Host Plant	Fungal Disease	Disease-Causing Fungi	Symptoms	References
Banana	Panama wilt	*Fusarium oxysporum* f. sp. *cubense*	Older leaves turn yellow, then necrotic and collapse, with vascular bundles exhibiting purplish brown discoloration	[115]
	Anthracnose	*Colletotrichum musae*	Brown lesions on ripened banana fruits, mature lesions become diamond-shaped, dark brown to black color with yellow halos	[115,116]
	Banana black Sigatoka (black leaf streak)	*Mycosphaerella fijiensis*	Reddish brown spots on the lower leaf surface that later develop into large spots	[115]
	Cordana leaf spot	*Cordana musae*	Pale brown, necrotic, oval-shaped spots on leaves, characterized by concentric zonation with a dark margin surrounded by a yellow halo	
Bean	Anthracnose	*Colletotrichum lindemuthianum*	Dark brown necrotic lesions on leaves accompanied by reduced photosynthetic activity	[62]
	White mold	*Sclerotinia* sclerotium	Water-soaked, circular, dark green lesions on leaves, pods, stems, and branches, with white cottony mycelium growing on infected flower surfaces	[117]
	Angular leaf spot	*Pseudocercospora griseola*	Angular-shaped necrotic lesions with yellow halos on leaves, reddish brown to black circular spots on pods, elongated, brown lesions on stems, petioles, and branches	[118]
	Powdery mildew	*Erysiphe polygonii*	Leaves covered with whitish grey powdery growth, later infected leaves turn yellow and drop off, infected pods and stems covered with white mildew	[119]
	Cercospora leaf spot	*Cercospora cruenta*, *C. canescens*	Defoliation and severe leaf spotting during pod formation and flowering	[120]
	Bean rust	*Uromyces appendiculaters*	White/light green spots on upper and lower leaf surfaces that turn into reddish brown pustules surrounded by yellow tissue, later leaves exhibit yellowing, senescence, and drop off	[121]
Coffee	Leaf rust	*Hemileia vastatrix*	Light yellow, small spots on upper leaf surface, orange-yellow to red-orange powdery lesions on underside of leaves, premature leaf drop	[122]
	Wilt disease	*Fusarium xylarioides*	Yellowing and withering of leaves, development of brown necrotic lesions, curling, drying, and falling of leaves	
	Cercospora blotch	*Cercospora coffeicola*	Round/irregular, small light brown to brown lesions with dark purple/black margins on leaves, brown, sunken, longitudinal/oval/irregular lesions with a grey center on green berries	
Maize	Anthracnose stalk rot	*Colletotrichum graminicola*	Foliar leaf blight, light brown spindle-shaped/oval-shaped water-soaked lesions with dark brown/purple margins on lower leaves, soft stalks with rot and reflective black strips on internodes	[123]
	Charcoal rot	*Macrophomina phaseolina*	Progressive wilting, premature dying, loss of vigor, decreased yield	
	Corn smut	*Ustilago maydis*	Formation of mushroom-like galls on maize kernels, chlorosis, decreased growth	
	Southern leaf blight	*Bipolaris maydis*	Cob rot, premature falling of corn ears	
Mango	Malformation disease	*Fusarium moniliformae* var. *subglutinans*	Presence of shorter, thicker, and highly branched inflorescences, increases in male flowers and reduces fertility in hermaphroditic flowers	[124]
	Powdery mildew	*Oidium mangiferae*	White powdery growth covering the stalks of the leaves, young fruits, inflorescences, and flowers	[125]
	Anthracnose	*Colletotrichum gloeosporioides*	Dark brown irregular or oval sunken spots on leaves, black necrotic sunken lesions on the peel of the fruit	[59]
	Dieback	*Lasiodiplodia theobromae*	Dark patches on green twigs, later complete defoliation result in fire scorch	[125]
	Stem end rot	*Alternaria alternata, Botryosphaeria* spp., *Botrytis cinerea*, *Colletotrichum gloeosporioides*, *Cytosphaera mangifera*, *Dothiorella mangiferae*, *Lasiodiplodia theobromae*, *Phomopsis mangiferae*, *Pestalotiopsis mangiferae*	Soft brown rot at the stem end of mango fruit that quickly spreads to the whole fruit, with secretion of straw-colored fluid from the stem end	[126]
Potato	Black dot	*Colletotrichum coccodes*	Macroscopic black sclerotia are the major symptom, later turns into brown or silver lesions	[127]
	Late blight	*Phytophthora infestans*	Irregular-shaped, water-soaked, pale green lesions near the margins and tips of leaves; they grow rapidly and turn into brown to purplish black, large, necrotic lesions; later the whole crop becomes blackened blight	[128]
	Grey mold	*Botrytis cinerea*	Tan-colored, wedge-shaped lesions on leaves; slimy brown rot on infected stems; discolored, pitted, sunken areas in tubers; grey color fuzzy growth on infected tubers	[129]
Rice	Sheath blight	*Rhizoctonia solani*	Oval/irregular/elliptical greenish grey spots on the leaf sheath; brown/dark brown sclerotia and brown silky mycelium loosely attached to the lesions in moist conditions; the entire leaf and plant later die	[130]
	Rice blast disease	*Pyricularia oryzae*	Elliptical spots with pointed ends featuring whitish/grey color centers and brown/reddish brown margins	[131]
	Brown spot	*Bipolaris oryzae*	Brown, small, circular/oval-shaped spots with whitish grey centers and yellow halos all over the leaf surfaces; small, dark brown/purple-brown young spots	[132]
Tea	Blister blight	*Exobasidium vexans*	Lemon green translucent spots on the first/second leaves, powdery white coating on blisters, shoot dieback	[133]
	Anthracnose	*Colletotrichum camelliae*	Yellowish green, small, diffuse spots, spots turn into dark brown, necrotic lesions with concentric rings, twig dieback	
	Gray blight	*Pestalotiopsis longiseta*	Concentric brown spots in the middle of the leaf, spots turn gray with brown margins and cover entire leaf, young shoot dieback	
Tomato	Damping-off	*Pythium* spp.	Complete rotting of seedlings, water-soaked and soft collar tissues	[134]
	Powdery mildew	*Leveillula taurica*	Light green to bright yellow spots on the upper surfaces of leaves, light powdery coatings on the lower leaf surfaces, necrotic lesions, defoliation, small and sunburned fruits	
	Tomato wilt	*Fusarium oxysporum* f. sp. *lycopersici*	Yellowing of leaves, downward curling, browning, and drying of infected leaves	
	Anthracnose	*Colletotrichum coccodes*	Small, circular, and sunken lesions on ripening fruit surfaces, spots enlarge into bruise-like depressions with a water-soaked appearance	
Wheat	Leaf rust	*Puccinia triticina*	Circular to slightly oval, non-merged pustules on stems or leaves	[135]
	Powdery mildew	*Blumeria graminis*	Greyish powder on stems and upper and lower surfaces of leaves	
	Crown root rot	*Bipolaris sorokiniana*	Infected crowns turn brown with brown to black small lesions on primary and secondary roots	

## 5. Management of Plant Fungal Diseases

Controlling fungal diseases before they appear is vital because of their severe impact on crop production. Farmers have long recognized the importance of cultural practices as a key component of integrated disease management systems. The aims of cultural control include minimizing contact with pathogens, creating unfavorable conditions for their growth, and reducing the available pathogen inoculum for infecting crop plants [14]. Cultural practices often act preventatively and indirectly against infections [136]. Techniques such as crop rotation, sanitation, tillage, enhancing crop growth conditions, and choosing resistant cultivars are among the cultural control methods [14]. The application of resistance cultivars, especially for diseases that are challenging to control using other methods, is one of the most effective strategies for managing diseases in many crops. Continual plant breeding programs are frequently required to minimize the emergence of novel pathogen races and the breakdown of host resistance. Therefore, it is crucial for growers to stay aware of the development of new cultivars and to have the capacity to evaluate their potential alongside other agronomic traits [136].

Some disease-causing pathogens can survive in the soil from one growing season to the next, often in the form of hyphae, sclerotia, or spores. When the same crop is repeatedly planted in the same area, any soil-borne pathogens associated with that crop can proliferate, leading to an increase in population levels over time. Crop rotation contributes to weed and pathogen management, while simultaneously improving soil fertility, moisture, and texture. Pathogens such as the *Colletotrichum* species, *Gaeumannomyces graminis*, *Phoma* species, and *Pyrenophora tritici-repentis* are among those most effectively controlled through rotation practices [14,136]. A well-nourished plant can withstand infections better than a plant with nutrient deficiencies or excessive fertilization. For example, phosphate fertilization can reduce the severity of disease caused by *Gaeumannomyces graminis* and inhibit the onset of take-all infection in barley, as well as minimize the incidence of potato scab caused by *Streptomyces scabies* [14]. Even though cultural practices offer a sustainable and effective approach to controlling fungal infections, there are also some negative impacts associated with them. For example, burning as a cultural practice may result in increased soil erosion and loss of nutrients in the soil [137].

Investigation into diseases causing economic losses and pathogen epidemiology have led to the development of principles for disease management. Therefore, synthetic fungicides have long been used for controlling fungal pathogens due to their effectiveness against plant fungal diseases [138]. Fungicides serve various purposes, including killing, preventing, repelling, or mitigating fungal pathogens. They are categorized based on methods of protection, such as preventive, anti-sporulant, and curative fungicides. Preventative fungicides can prevent the occurrence of infection, while anti-sporulant fungicides aim to prevent spore formation. Similarly, curative fungicides can inhibit the development of the disease after infection [139]. Fungicide mixtures, which comprise two or more fungicides in a single treatment are widely regarded as the most popular, well-researched, and recommended method for controlling plant pathogens [140]. The application of fungicides is increasing due to various factors. Fungicides are considered essential to global food security, particularly in changing climatic conditions, the spread of invasive fungal pathogens, and the development of resistance to fungicides [141]. Resistance to fungicides is a naturally occurring, inheritable change in the ability of a population to withstand plant-protection treatments that usually provide effective control. Instances such as *Penicillium digitatum* in citrus exhibiting resistance to sodium-o-phenylacetate and diphenyl, as well as *Tilletia foetida* developing resistance to hexachlorobenzene in Australia [142] are some examples of reported cases of fungicide resistance. These are relatively limited in number and not considered highly economically significant. Some advantages of using synthetic fungicides include providing highly effective control of fungal pathogens and offering a cost-effective approach [142].

Long-term and improper use of synthetic fungicides can have negative impacts due to their high tendency to remain in the environment and low biodegradability. This can lead to environmental pollution, resulting in chronic human diseases, ozone layer depletion, residual toxicity, and harm to non-target organisms [138,143]. According to the annual report by the European Food Safety Authority (EFSA), which investigated vegetables and fruits from 27 countries for pesticide contamination, dithiocarbamates are among the most common residual toxins [144]. Fungicides have been associated with several adverse effects, including dermatological, carcinogenic, neurological, and gastrointestinal impacts. Chronic health conditions attributed to fungicide application include cancers, birth defects, genetic disorders, tumors, blood disorders, and damage to the brain and nervous system [145]. The widespread application of fungicides can lead to the development of fungicide resistance due to the potential for genetic mutations in pathogens [146]. For example, current synthetic fungicides have been found to be ineffective against anthracnose, wheat take-all, vascular wilt, and other root-infecting pathogens [147]. Fungal species such as *Venturia inequalis*, *Phytophthora infestans*, *Colletotrichum musae*, *Colletotrichum gleosporioides*, *Diplodia natalensis*, and *Phomopsis citri* have exhibited resistance to specific fungicides such as dodine, metalaxyl, benomyl, and benzimidazole [146].

Due to the adverse effects of chemical fungicides, there is a growing demand for novel fungicides that have a low toxicity and are sustainable and safer [138]. Microbial metabolites offer a promising alternative to fungicides, showing an extensive range of biological and chemical benefits. Microorganisms have the potential to produce secondary metabolites with diverse biological functions. As a result, there is an increased possibility of discovering novel antifungal compounds that either act in a new manner or do not exhibit negative interactions associated with current fungicides [147]. Blasticidin S was the first microbial fungicide available and was utilized to control rice blast disease caused by *Magnaporthe grisea* (=*Pyricularia grisea*). This nucleoside fungicide is derived from the metabolites of *Streptomyces griseochromogenes*. Another microbial metabolite Validamycin A, produced by *Streptomyces hygroscopicus* var. *limoneus*, has demonstrated effectiveness in controlling rice sheath blight caused by *Rhizoctonia solani* [138].

## 6. Antagonistic Activity of Endophytic Fungal Species

The ability of endophytic fungi to hinder or suppress the growth and function of other microbes is a key component of their antagonistic nature. Antibiosis is primarily characterized by antagonistic strains releasing metabolic products that inhibit the growth of pathogenic fungi. It is one of the most significant methods of controlling plant pathogens by endophytic fungi, in which the antagonists produce a wide range of secondary metabolites, including toxins and antibiotics against pathogens [148,149]. Antibiosis is not always linked with the growth rate of the antagonistic strain [150]. Antagonists employ a range of direct and indirect mechanisms to control pathogenic diseases, each contributing to the suppression of pathogens in unique ways. Direct interactions include antibiosis, where antagonists produce antibiotics or secondary metabolites that inhibit pathogen growth [8,23]. Mycoparasitism represents another direct approach, involving antagonists that derive some or all their nutrients from the host of a fungal pathogen [18,23]. Antagonists can activate induced resistance mechanisms in plants, thereby enhancing their defensive capacity against fungal pathogens [18]. The secretion of extracellular hydrolytic enzymes by antagonists can break down the cell walls of pathogens, further limiting their virulence [8]. Endophytic fungi can increase the host plant defense by inducing the formation of various structural defense mechanisms, including cellular, cytoplasmic, and histological barriers, formation of callose, tyloses, abscission and cork layers, and the deposition of gums [18,23,43]. Biochemically, endophytic fungi elicit plant defense mechanisms through the production of phenolic compounds, proteins, and by triggering hypersensitive responses. Additionally, they stimulate the production of phytohormones such as salicylic acid and jasmonic acid, which are central defense signaling molecules, as a long-lasting defense response to pathogens [18,23]. Salicylic acid mediates the systemic acquired resistance against pathogens and promotes the accumulation of pathogenesis-related proteins, thereby reinforcing cell wall boundaries and directly lysing invading cells [23]. For example, *Trichoderma asperellum* induces cell wall-degrading and defense-related enzymes against leaf spot fungi in lettuce (*Lactuca sativa*) [18]. Similarly, *Fusarium solani*, an endophytic fungus in tomato roots, induces systemic resistance against the foliar pathogenic fungus *Septoria lycopersici* by activating pathogenesis-related genes [23].

Another mechanism involves the detoxification of virulence factors produced by pathogens, neutralizing their ability to infect host plants [8,39]. Indirectly, antagonists can outcompete pathogens for space and nutrients thus starving them and reducing their proliferation. The production of plant growth-enhancing hormones such as gibberellic acid and indole-3-acetic acid (IAA) by antagonists improves plant health and supports the natural defenses of a plant against pathogenic invasion [8,18,23]. These mechanisms can collectively contribute to the effective management of some plant pathogenic fungi [8,18].

The potential for applying endophytic fungi as biocontrol agents against plant pathogens has highly expanded due to their unique characteristics. For instance, some of these fungi exhibit high reproductive rates both sexually and asexually, being target-specific organisms, have short generation times, and can survive even in the absence of a host by transitioning from parasitism to saprotrophic mode [8]. Antagonistic fungi display higher host specificity and rapid mass synthesis, often with negligible effects on non-target species. To effectively apply antagonistic fungi as biocontrol agents, it is essential to understand the mechanisms underlying their impact on plant diseases [151]. However, this has led to significant research regarding the application of secondary metabolites from endophytic fungi as antimicrobial agents. Consequently, commercial agricultural products containing microbial biocontrol agents have been successfully utilized in modern sustainable agriculture [8].

Mildew and rust-like diseases caused by biotrophic fungal pathogens often exhibit a brief epiphytic phase and minimal reliance on external nutrients for penetration. Mycoparasitism appears to be a more effective bio-control strategy against these biotrophic pathogens during the epiphytic phase. Nutrient competitor antagonists are potentially useful against *Alternaria*, *Botrytis*, *Phoma*, and *Septoria* species, resembling unspecialized necrotrophs. They grow saprophytically on the phylloplane and absorb external nutrients before attacking. However, their primary mode of action is recognized as nutrient competition rather than through antibiosis [18].

### 6.1. Antagonistic Activity of Trichoderma spp.

Research studies have shown that the *Trichoderma* species can control plant diseases including soil-borne, panicle, and certain leaf diseases [152]. *Trichoderma* has been recognized for approximately seven decades for its parasitizing or antagonistic effect on other pathogenic fungi [151]. The selection of *Trichoderma* as an antagonist is effective for many reasons, i.e., the ability to survive even under unfavorable conditions, high reproductive capacity, the ability to alter the rhizosphere, intense aggressiveness towards plant pathogenic fungi, the capability of promoting plant growth, and the potential to enhance the efficiency of nutrient utilization [151,152]. *Trichoderma* species are utilized in agriculture to control phytopathogens based on various mechanisms of action, including mycoparasitism, competition, antibiosis, damaging, and coiling around pathogen hyphae and the formation of volatile chemical compounds [153,154,155,156]. It is one of the most successful bio-fungicides used in modern agriculture, comprising nearly 60% of registered products worldwide. Additionally, they produce secondary metabolites that stimulate plant defense mechanisms in canola, tomato, and pea [36].

Different studies have shown the efficacy of *Trichoderma* spp. in controlling plant fungal pathogens, such as *Botrytis cinerea*, *Collectotrichum* spp., *Fusarium oxysporum*, *Pseudocercospora* spp., *Pythium ultimum*, *Rhizoctonia solani*, and *Sclerotinia sclerotiorum*. Specifically, *Trichoderma harzianum* and *T. viride* have been shown to hinder the negative effects of plant fungal pathogens belonging to 18 genera, including *Botrytis*, *Fusarium*, and Rhizoctonia [152]. Al-Askar et al. [157] reported that *Trichoderma asperellum* endophytic fungus isolated from decayed maize stover exhibited the capability to suppress Fusarium wilt disease in tomatoes, which is caused by *Fusarium oxysporum* f. sp. *lycopersici* through the production of citric acid as a biocontrol metabolite. Additionally, a strain of *Trichoderma gamsii* has shown the ability of suppressing *Fusarium culmorum* and *F. graminearum*, causing FHB in rice [158]. Sornakili et al. [159] illustrated that *Trichoderma longibrachiatum* exhibits metabolite-induced antagonistic activity against phytopathogens in rice, including *Macrophomina phaseolina*, *Magnaporthe grisea*, and *Rhizoctonia solani*. Its antagonistic behavior is attributed to the secretion of hydrolytic enzymes, mycoparasitism, and the production of volatile organic compounds (VOC) [159].

Black point infection caused by *Alternaria*, *Bipolaris*, *Drechslera*, and *Fusarium* species is responsible for approximately 24% of economic loss in wheat. Among the above-mentioned pathogens, *Alternaria alternata* showed the highest inhibition, while *Drechslera halodes* showed the lowest inhibition by the *Trichoderma harzianum* and *T*. *viride* endophytes. Predominant bioactive components responsible for the above antagonistic activity were due to the cyclooctanol in *Trichoderma viride* and 6-pentyl-pyrone in *T*. *harzianum*. Furthermore, *Trichoderma viride* showed strong antagonistic activity against carbendazim-resistant fungal phytopathogen strains, causing black point disease in wheat [160]. Under greenhouse conditions, *Trichoderma harzianum* wheat-bran inoculum significantly reduced disease incidence in beans caused by *Sclerotium rolfsii* (97% disease reduction) and *Rhizoctonia solani* (57% disease reduction), either individually or in combination. The wheat-bran inoculum of *Trichoderma harzianum* was also effective in decreasing disease incidences in field experiments with beans, tomatoes, and cotton, and resulted in a significant improvement in the yield of beans [161].

*Trichoderma harzianum* and *T*. *viride* also showed significant antagonistic activity by inhibiting the mycelial growth of *Fusarium proliferatum* and *F*. *verticillioides*, which cause stalk rot infection in maize [162]. Degani et al. [163] illustrated that *Magnaporthiopsis maydis*, the causative agent of late wilt disease in maize, can be inhibited using the endophytic fungus *Trichoderma asperellum*. It produced a secondary metabolite 6-Pentyl-α-pyrone, which is a highly potent antifungal compound in controlling late wilt disease. Lopez-Lopez et al. [156] showed that the *Trichoderma* species isolated from avocado orchards can inhibit fungal infections in avocado plants. According to the results of the study, *Trichoderma harzianum* (TSMICH7) strains exhibited more than 80% inhibition against the following four phytopathogens: *Colletotrichum gloeosporioides* (causing anthracnose), *Diaporthe* spp. (causing stem end rot), *Neofusicoccum parvum* (causing soft rot) and *Phomopsis perseae* (causing fruit rot and phomopsis spot) in avocado. Zhang et al. [164] reported that *Trichoderma atroviride* (HN082102), a salt-tolerant marine endophytic fungus, exhibits antagonistic activity towards *Fusarium oxysporum*, which is the causative agent of root rot disease in cucumbers. Its antagonistic mechanism involves the production of volatile and non-volatile secondary metabolites to inhibit the mycelial growth of the pathogen. *Trichoderma* strains showed antagonistic effects against *Fusarium oxysporum* which is responsible for root rot infection in soybean through mycelial inhibition. These strains displayed beneficial mycoparasitism activity against *Fusarium oxysporum* by coiling around and penetrating the hyphae, as well as dissolving the cell walls of the target fungal species [165].

*Trichoderma koningiopsis* (PSU3-2 strains) displayed a significant antagonistic activity against anthracnose disease, caused by *Colletotrichum gloeosporioides* in chili pepper with multiple inhibition mechanisms. These mechanisms include producing antifungal metabolites to inhibit mycelial growth, generating cell wall-degrading enzymes, including chitinase and β-1,3-glucanase, distorting and lysing the shape of hyphae and reducing the lesion size [166]. *Fusarium oxysporum* f. sp. *cubense*, a soil-borne fungus causes significant economic loss in bananas by causing Fusarium wilt disease. To manage this outbreak, *Trichoderma reesei* (CSR-T-3 strain) was effectively used as a biocontrol endophyte. Biocontrol mechanisms of CSR-T-3 includes the production of antifungal compounds, mycoparasitism, chlamydospore production, and reduction in fungal toxins [167]. Additionally, *Trichoderma* strains T16 and T23 have been found to be effective against *Phakopsora pachyrhizi* which causes Asian bean rust disease. These strains produce potent antifungal secondary metabolites that inhibit the germination of uredospores of pathogenic fungal species [168]. *Trichoderma samuelsii*, an endophyte isolated from *Thymus mongolicus* Ronn (thyme) expressed antagonistic activity against black mold disease in goji berries caused by *Alternaria alternata* by producing VOCs such as 6-pentyl-2H-pyran-2-one [169].

**Figure 1:**
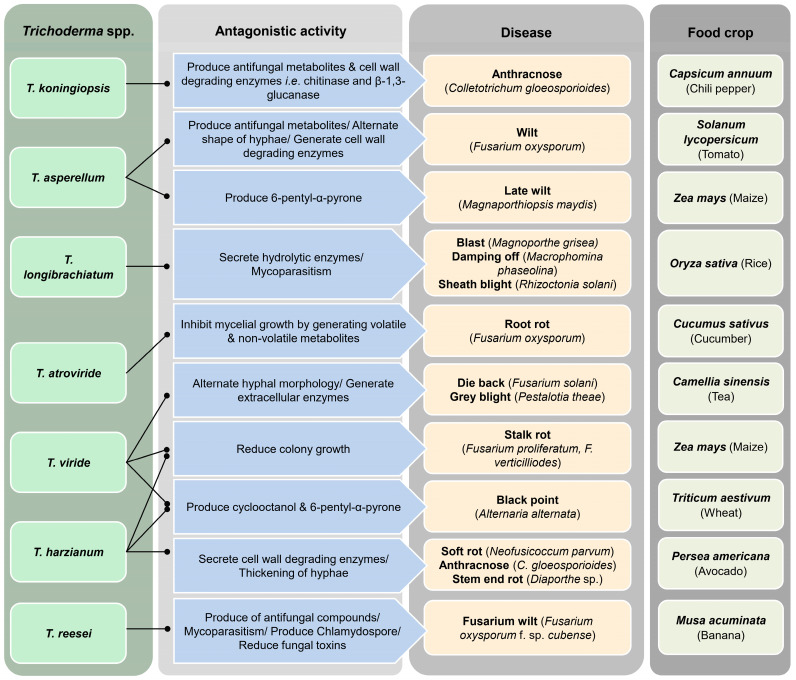
Antagonistic activity of various *Trichoderma* species against fungal pathogens [156,157,159,160,162,163,164,166,167,170].

*Fusarium solani* and *Pseudopestalotiopsis theae* (*Pestalotia theae*), which cause dieback disease and the grey blight disease in tea, are among the most devastating fungal pathogens [170,171,172]. *Trichoderma viride* (SDRLIN1 strain) that was isolated from tea rhizosphere soils showed significant antagonistic abilities against both diseases through multivariate inhibitory mechanisms. These mechanisms include the production of extracellular enzymes and alterations in the hyphal morphology, such as hyphal swelling, distortion, and cytoplasm aggregation [170]. Numerous *Trichoderma* species have been applied as biocontrol agents against various phytopathogenic fungal diseases. Figure 1 summarizes the key inhibitory actions of *Trichoderma* species against fungal pathogens in different plant species.

Recently, many *Trichoderma*-based commercial fungal biocontrol products have been developed by numerous companies around the globe. Binab T was the first registered commercial *Trichoderma* formulation for controlling plant diseases [173]. *Trichoderma harzianum* strain-22 produced by Bioworks, Geneva, Switzerland, and TGT Inc., New York, NY, USA; *T. viride* produced by Ecosense laboratories, Mumbai, India; *Trichoderma virens* produced by Grace-Sierra Co., Baltimore, MD, USA; and *Trichoderma parceramosum* produced by BioSpark Corporation in Laguna, Philippines, are among the major commercially available products of *Trichoderma* for biological control [18]. Dutta et al. [173] highlighted several examples of commercial *Trichoderma*-based products, including a combinatory formulation of *T*. *harzianum* and *T*. *viride* produced by Biotech International Ltd. India under the name Bioderma, a *Trichoderma viride* formulation from Poland named Bip T, and a *T*. *harzianum* formulation known as Plant Shield by Bioworks, Inc., USA.

### 6.2. Antagonistic Activity of Penicillium spp.

The *Penicillium* species have been noted for their antagonistic nature to protect host plants from pathogenic attacks through the production of antagonistic metabolites [174]. Due to their significant antagonistic potential, several *Penicillium* species have been extensively studied for their application in managing various phytopathogenic fungal infections. For example, these species are effective against diseases, such as *Cercospora beticola* which causes cercospora leaf spot in sugar beets, *Fusarium solani* which is responsible for root rot in okra, *Pyricularia oryzae* which causes rice blast disease, and *Macrophomina phaseolina* which causes charcoal rot in sorghum and mung bean. Among them, some *Penicillium* species have gained considerable attention for their antagonistic behavior against pathogens, achieved by producing antibiotics and inducing host resistance through various defense mechanisms. The *Penicillium* species secrete a wide variety of bioactive metabolites, including IAA, siderophore, lipase, hydrocyanic acid, protease, and β-1,3 glucanase, which contribute to disease suppression and iron absorption in plants [175].

The strain *Penicillium oxalicum* (PO212) has demonstrated efficacy as a biocontrol agent in managing a variety of fungal diseases in crops across different environments, including growth chambers, glasshouses, and fields [176,177,178,179]. *Penicillium oxalicum* suppressed pathogens by secreting extracellular lytic enzymes, including chitinases, α-1,3-glucanases, and cellulases [154]. The application of *Penicillium funiculosum* has effectively managed bark infections on citrus plants, such as orange seedlings and lemon trees, and has been employed against Phytophthora root rot in citrus [180]. The *Penicillium* species isolated from the rhizosphere were found to control black rot disease in onions (*Allium cepa*), which is caused by *Aspergillus niger* [179].

**Figure 2:**
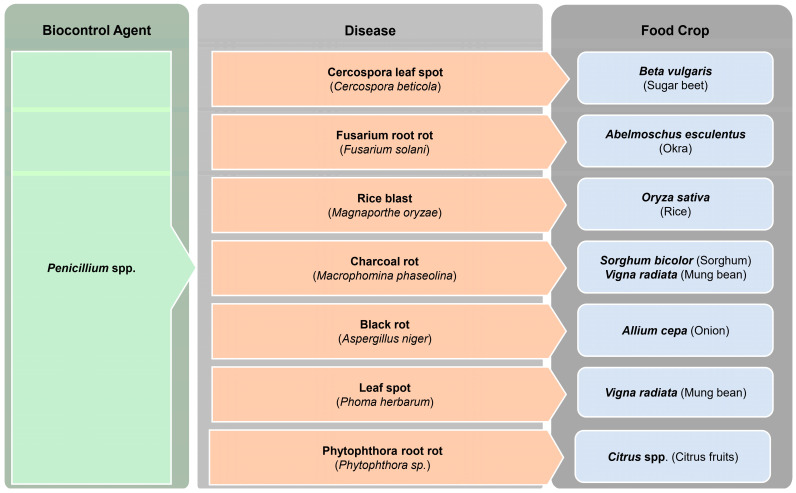
Antagonistic activity of *Penicillium* spp. against fungal pathogens in various food crops [175,179,180].

*Penicillium citrinum* (BTF08 strain), isolated from wild banana plantlets, has shown promising antagonistic activity against Fusarium wilt in bananas, caused by *Fusarium oxysporum* f. sp. *cubense* race 4 [181]. These strains induce host resistance by producing important biochemical markers, such as peroxidase, phenylalanine ammonia-lyase, and polyphenol oxidase [181]. Furthermore, various *Penicillium* species, i.e., *P. crustosum*, *P. digitatum, P. janczewskii, P. oxalicum*, and *P. verrucosum* have been effective in reducing dry biomass production of *Phoma herbarum*, a pathogen which causes leaf spot disease in mung beans. Among them, *Penicillium janczewskii* showed the most significant inhibition [175]. Figure 2 summarizes various fungal diseases in food crops that are effectively controlled by *Penicillium* spp.

### 6.3. Antagonistic Activity of Muscodor spp.

**Figure 3:**
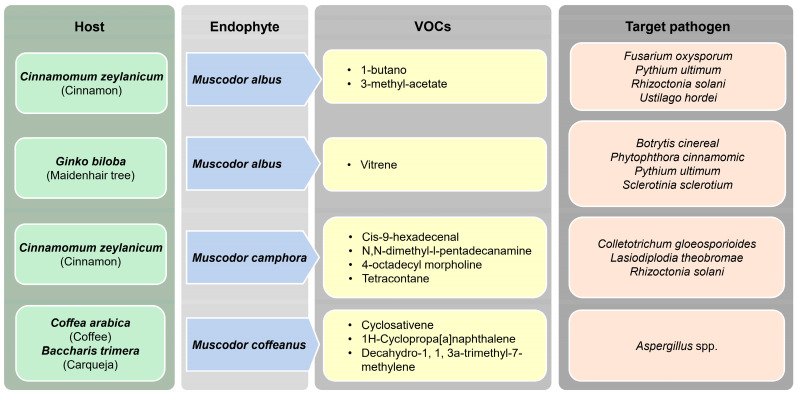
Different Volatile Organic Compounds effective against controlling phytopathogens produced by *Muscodor* spp. [186,187,188,189,190].

The genus *Muscodor* is recognized as an efficient endophytic fungus known for synthesizing various VOCs with antimicrobial properties, which makes it a potential mycofumigant [182,183,184]. *Muscodor* is a widespread endophytic fungus found in tropical gramineous plants, and numerous strains have confirmed the potential to inhibit or kill a variety of phytopathogens [185]. Strobel et al. [186] discovered that *Muscodor albus,* isolated from *Cinnamomum zeylanicum* is an efficient mycofumigant, which produces a mixture of VOCs. Their study identified 1-butanol and 3-methyl-acetate esters as the most effective inhibitory compounds, which impact a range of plant pathogenic fungi, including *Fusarium oxysporum*, *Pythium ultimum*, *Rhizoctonia solani*, and *Ustilago hordei* [186,187]. *Muscodor albus* strain GBA, isolated from *Ginko biloba* plants, showed higher effectiveness in inhibiting *Botrytis cinerea, Phytophthora cinnamomic, Pythium ultimum*, and *Sclerotinia sclerotium* by achieving 100% mortality after exposure [188]. This fungal strain uniquely produced vitrene terpenoid, which was not observed in other *Muscodor albus* strains [188]. Meshram et al. [189] showed the anti-fungal activity of *Muscodor camphora*, isolated from *Cinnamomum camphora* plants. The VOCs produced by this endophyte included cis-9-hexadecenal, N,N-dimethyl-l-pentadecanamine, 4-octadecyl morpholine, and tetracontane, showing a 13–70% growth inhibition of various fungal pathogens, i.e., *Colletotrichum gloeosporioides, Lasiodiplodia theobromae*, and *Rhizoctonia solani* [189]. *Induratia coffena* (*Muscodor coffeanus*) isolated from *Coffea arabica* (coffee plants) and *Baccharis trimera* (carqueja) showed high efficacy in suppressing or killing *Aspergillus* spp., apart from the inhibition of *Aspergillus ochraceus* inoculated into coffee beans [190]. *Induratia coffeana* and *I*. *yucatanensis* (*M*. *yucatanensis*) isolated from coffee plants produced VOCs that can inhibit the pathogens of anthracnose (*Colletotrichum lindemuthianum*), white-grey mold (*Sclerotinia sclerotiorum*), and angular leaf spot diseases (*Pseudocercospora griseola*) in beans [184]. Certain species of *Muscodor* are commercially available as biocontrol agents [183]. Ennoble^TM^ is a commercially available mycofumigant product derived from the *Muscodor albus* SA-13 isolate, developed by Marrone Bio Innovations (MBI). This product serves as a replacement for methyl bromide in agriculture, and it is effective against plant pathogenic fungi such as *Pythium ultimum*, *Phytophthora capsici*, *Rhizoctonia solani*, *Tilletia caries*, and *Verticillium dahlia* [191]. *Muscodor* is used as a soil fumigant to control soil-borne diseases, including root rot and damping-off in plants [189]. Figure 3 provides an overview of the various VOCs produced by *Muscodor* spp. that help in controlling several fungal pathogens.

### 6.4. Antagonistic Activity of Other Endophytic Fungi

Researchers continue to investigate and discover new species of endophytes with novel approaches to inhibit phytopathogens. *Fusarium* spp. is typically known as plant pathogenic fungi associated with root wilts and root rots. Interestingly, some studies have shown that certain species of *Fusarium* can serve as biocontrol agents against various phytopathogens [192]. Sawai et al. [193] reported that *Fusarium solani* can act as an antagonistic fungal species against *Valsa ceratosperma*, the causal agent of Japanese apple canker disease. Zhao et al. [150] isolated the endophyte *Talaromyces trachyspermus* (R-17 strain) from the medicinal plant *Cornus officinalis* and showed its antifungal activity against crown rot disease in wheat by synthesizing polyketides and peptides to inhibit the mycelial growth. *Monosporascus cannonballus* is a crucial phytopathogenic fungus responsible for vine decline disease and root rot infection in muskmelon fruit. Researchers discovered that several endophytic fungal species isolated from Shirazi Thyme (*Zataria multiflora*), a medicinal plant, can inhibit the mycelial growth of *Monosporascus cannonballus* in muskmelon. These endophytes inhibited the mycelial growth of *Monosporascus cannonballus* by inducing morphological abnormalities, such as shrinkage, turgidity, and disintegration [194].

*Colletotrichum acutatum* is a fungal species responsible for blight and anthracnose in important host plants, such as almonds, citrus, peaches, olives, and strawberries. Fungal species isolated from olive (*Olea europaea* cv. *Galega vulgar*) leaves, including *Alternaria*, *Arthrinium*, *Aspergillus*, *Epicoccum*, *Fusarium*, and *Nigrospora* species exhibited different inhibitory mechanisms, including producing antifungal VOC and competition [195]. Demirci et al. [196] discovered that *Acremonium* sp., *Gliocladium viride*, *Paecilomyces sulphurellus*, *P*. *marquandii*, *Penicillium camemberti*, *P*. *frequentans*, *P*. *expansum*, *P*. *nigricans*, *P*. *phialosporum*, *P*. *olsonii*, *Sporothrix schenckii*, and *Verticillium dahliae* isolates exhibited strong antagonistic activity against *Rhizoctonia solani*, which is responsible for black scurf and stem canker infections in potatoes. The application of specific strains of *Pichia anomala*, particularly Moh 93 and 104, significantly reduced Diplodia rot disease in guava that is caused by *Botryodiplodia theobromae* [197].

*Cladosporium cladosporioides* is identified as an antagonistic endophyte capable of suppressing the mycelial growth of rice pathogens, especially in cases of rice blast disease [198]. Additionally, *Epicoccum nigrum*, *Penicillium oxalicum* and *Trichoderma harzianum*, isolated from onion leaves displayed inhibition of purple blotch disease caused by *Alternaria porri* on onions. *Epicoccum nigrum* displayed inhibition through the production of non-toxic bioactive compounds, such as epirodins, avipin and epicorazines. Cosoveanu et al. [199] showed that endophytic fungi *Acremonium strictum*, *Alternaria* sp., *Aureo basidiumpullulans*, *Bionectria ochroleuca* and *Chaetomium spirochaete* isolated from grapevine (*Vitis vinifera*) plants can suppress the fungal pathogen *Botrytis cinerea*, which is the causative agent of Botrytis rot disease in grapes.

*Curvularia lunata*, the causal agent of dirty panicle disease in rice, *Fusarium moniliforme*, the causal agent of bakanae disease in rice, stalk rot disease in corn, red rot disease in sugarcane, and *Rhizoctonia solani*, the causal agent of sheath blight disease in rice plants, have also shown suppression due to endophytic fungi. Endophytic fungus *Wickerhamomyces anomalus* isolated from leaf tissues of rice, corn, and sugarcane have the potential to inhibit the above pathogens, *Curvularia lunata*, *Fusarium moniliforme*, and *Rhizoctonia solani* by producing VOCs, cell wall-degrading enzymes, siderophores, and solubilizing phosphates and zinc oxide [200].

Tchamgoue et al. [201] showed that endophytic fungi *Botryosphaeria*, *Phomopsis,* and *Xylaria* species isolated from guava (*Psidium guajava*) plants suppress *Fusarium oxysporum* f. sp. *cubense*, which causes Panama disease, and *Mycosphaerella fijiensis*, which causes black leaf streak (Sigatoka) disease in bananas. Those endophytes showed various antagonistic mechanisms, such as producing bioactive metabolites, competition for space and nutrients, and mycoparasitism. Lugtenberg et al. [202] discovered *Daldinia concentrica*, an endophytic fungus found in Israeli olive trees, capable of producing over 28 VOCs with potential applications in post-harvest management of phytopathogens. Further information on the antagonistic activity of various endophytic fungi against phytopathogens in food crops, along with their mechanisms of antagonism, is provided in Supplementary Table S1.

Simamora et al. [203] showed that certain species of *Aspergillus*, *Fusarium*, and *Ramichloridium*, isolated as endophytes from cocoa plants, exhibit inhibitory activity against *Phytophthora palmivora*, the pathogenic fungus of cocoa black rot pod disease. These endophytes exhibited a combination of inhibitory mechanisms, including competition, antibiosis, and mycoparasitism [203]. According to Putri et al. [204], *Curvularia chiangmaiensis*, *Fusarium solani*, and *Trichoderma asperellum* isolated from rice leaves showed the highest inhibition against *Pyricularia oryzae*, the causative agent of rice blast disease. These endophytes produced hydrolytic enzymes such as chitinase and cellulase to degrade the cell walls of pathogens [204]. The endophytic *Alternaria*, *Chaetomium*, *Daldinia*, and *Rosellinia* species isolated from mango leaves have been recognized as biocontrol agents against *Botrytis cinerea* (causal agent of grey mold disease) and *Penicillium digitatum* (causal agent of green mold disease) in various food crops. *Chaetomium* species exhibited antibiosis, mycoparasitism, induction of defense responses, and competition as their biocontrol mechanisms, while the *Daldinia* species showed competition for nutrients and space as their antagonistic activity against pathogens [205]. *Aspergillus terreus* isolated as an endophyte from *Phaseolus vulgaris* (common bean) showed effective biocontrol activity against *Rhizoctonia solani*, causing damping-off disease in *Phaseolus vulgaris* and *Vicia faba* cereal crops. It stimulated the production of organic compounds responsible for plant defense systems such as antioxidant enzymes, phenols, and prolines [206]. Sopialena and Sofian [207] reported that *Aspergillus* and *Gliocladium* isolated from *Amorphophallus muelleri* (porang plants) could suppress the pathogenic *Fusarium* species, which causes bulb rotten disease, and *Colletotrichum gloeosporioides*, which causes anthracnose disease in porang plants. These endophytes used various inhibitory mechanisms, including antibiosis, competition, and mycoparasitism [207].

*Pochonia chlamydosporia* endophytic fungi isolated from *Dolichos lablab* (lablab beans) exhibited a biocontrol effect against *Fusarium oxysporum* f.sp. *cubense*, the causative agent of fusarium wilt disease in banana plants [208]. The *Paecilomyces* endophytic strain isolated from *Moringa oleifera* (drumstick tree) leaves exhibited antagonistic activity against *Rhizoctonia solani*, the pathogenic fungus that causes black scurf and stem canker diseases in potatoes. These endophytes produced fungistatic secondary metabolites such as methyl esters and octadecenoic acid to suppress *Rhizoctonia solani* [209]. Yabaneri and Sevim [210] showed that *Ophiognomonia leptostyla*, the pathogen causing anthracnose disease in walnut (*Juglans regia*) can be inhibited by applying *Alternaria* sp. CC-3, an endophytic fungus isolated from walnut plants. *Acremonium* sp. Ld-03, isolated from *Lilium davidii* (lily) bulbs, showed antifungal activity against phytopathogenic fungi *Botryosphaeria dothidea*, *Botrytis cinerea*, *Fusarium oxysporum*, and *F*. *fujikuroi*. This endophyte produced siderophore and secondary metabolites, including xanthurenic acid, gancidin W, valyl aspartic acid and peptides as inhibitory mechanisms [211]. Attia et al. [212] reported that *Aspergillus terreus* (ON380424), an endophytic fungus isolated from *Ocimum basilicum* (basil) leaves, can suppress *Alternaria solani*, the pathogenic fungus which causes early blight disease in eggplants. The inhibition involved a combination of mechanisms, including the induction of systemic resistance, reduction in oxidative stress, and enhancement of photosynthetic pigments, phenolic compounds, and antioxidant enzymes [212]. *Xylaria adscendens*, an endophytic fungus isolated from Tahiti lime (*Citrus citrus* × *latifolia*) plants exhibited antagonistic activity against *Colletotrichum acutatum*, the causative agent of lime anthracnose disease [213]. Munoz-Guerrero et al. [213] suggest that these endophytes employed competition, antibiosis, or mycoparasitism for their inhibitory effects.

The oomycete *Pythium oligandrum* functions as a mycoparasitic biocontrol agent, capable of antagonizing various plant pathogens and enhancing plant growth [214]. It is also an endophyte capable of colonizing the root rhizosphere of many crop plants [215]. Studies have shown that *Pythium oligandrum* can suppress *Fusarium culmorum*, *F*. *graminearum* and *F*. *oxysporum* in wheat, barley, and tomatoes [216,217,218,219]. A plant defense elicitor oligandrin produced by *Pythium oligandrum* endophytes has shown the potential to inhibit the progression of *Botrytis cinerea*, which is responsible for gray mold disease on grapevine leaves. In response to oligandrin treatment, grapevine plants exhibited modifications in cuticle thickness and accumulation of phenolic compounds [220]. Gerbore et al. [221] identified that the culture filtrate from *Pythium oligandrum* (oligandrin) is effective in controlling *Erysiphe necator* on grapevines [221].

## 7. Future Prospects

Based on the available literature, a wide range of endophytic fungi show the potential to be used as disease-controlling agents, especially in food crops. These vigorously effective endophytes often share common traits, such as the production of growth-inhibiting enzymes, antimicrobial agents, and compete for nutrients and space with phytopathogens. Regardless of the numerous endophytic fungi showing potent antagonistic activity against various phytopathogens, the *Trichoderma* species has received particularly great attention in this field. Their efficacy and potential for development into sprayable inoculants resembling chemical pesticides facilitate their easy incorporation into integrated strategies for controlling phytopathogens [23].

While researchers continue to explore novel approaches and applications of endophytic fungi as antagonists, challenges remain in their commercial-scale applications. To succeed as commercial products, they should fulfil farmer requirements, including realistic prices, ease of application, consistent positive results, and long shelf life [172]. One significant challenge is the high cost associated with commercializing biological control agents. This cost arises from various processes, i.e., the isolation of the microorganism in pure culture or enrichment, detection and characterization, formulation development, mass production, efficacy evaluation of the product, evaluation of storage stability, identification of manufacturing partners, consideration of challenges related to human and environmental safety, authorization procedures, and marketing efforts [8]. The field-level application of these fungal metabolites in agriculture for disease management has yet to be largely investigated, primarily because much of the available research is limited to laboratory or greenhouse experiments. In such a situation, there is a need to evaluate a majority of the reported organisms/metabolites under field experiment conditions before developing them as commercial biocontrol agents [190].

Large-scale commercial applications of these products require thorough examinations of the behavior of specific endophytic populations within their host plants. Additionally, the large-scale production of endophytes as commercial products remains limited due to the specificity of endophytes with their host plants [23]. Investigating the complex relationships between host plants and endophytic fungi is essential for bridging current knowledge gaps and understanding the broad spectrum of potential benefits they offer. Commercializing endophytes for controlling phytopathogens in food crops requires confirmation of their identity using morphological and molecular tools [8,220].

However, the sustainable agricultural industry is exploring different approaches to overcome these challenges and advance the utilization of endophytic fungi as biocontrol agents [8]. Future studies might also investigate the synthesis of endophytic nanoparticles capable of protecting plants [23]. Exploring the genes and traits associated with the relationships between endophytes and their hosts can be effectively achieved through the application of genetic analysis and enzyme activity measurement [8,220]. Traditional evaluations of endophytic fungi have primarily relied on culture-based methods and molecular identifications. However, next-generation sequencing methods have been recently utilized to gain a more comprehensive understanding of fungal diversity and its distribution. The advancements in biotechnology and genetic modification techniques have greatly contributed to the development of novel fungal strains with enhanced biocontrol capabilities and efficiency [8]. Genetic modification techniques can introduce novel features, such as improved metabolic regulation and phytoremediation capabilities [23].

## 8. Conclusions

Fungal diseases affecting food crops, including cereals, vegetables, and fruits, result in significant economic losses. Throughout history, farmers have used various methods to control fungal diseases, initially relying on conventional cultural practices and more recently turning to synthetic fungicides. The prolonged application and improper handling of synthetic fungicides have led to numerous negative impacts on the environment and human health. As a solution, there is a growing interest in using biological agents with sustainable agricultural prospects. Endophytic fungi, comprising a phylogenetically diverse group, colonize plant tissues asymptomatically and sporadically, forming saprophytic, mutualistic, or commensal interactions with their host plants. Their ability to synthesize bioactive compounds and promote plant growth has gained increased attention for their potential use as antagonists against phytopathogens. Endophytic fungi use multivariate direct or indirect mechanisms to control plant fungal pathogens. These include antibiosis, mycoparasitism, induction of plant resistance responses against fungal pathogens and the enhancement of plant growth through phytohormone production. Additionally, they reduce the adverse effects of fungal pathogens by secreting extracellular hydrolytic enzymes, detoxifying virulence factors, suppressing fungal mycelium growth, and competing for nutrients and space within the ecosystem. Additionally, endophytic fungal species are known to produce a variety of secondary metabolites that exhibit efficacy in controlling diverse fungal pathogens. Citric acid, cyclooctanol, and 6-pentyl-α-pyrone produced by *Trichoderma* spp. and 1-butanol, 3-methyl acetate, vitrene, and tetracontane produced by *Muscodor* spp. can be given as some examples of them. Consequently, many studies have highlighted the frequent use of *Trichoderma* spp. in controlling phytopathogens across various food crops. Moreover, numerous studies have also revealed that nonpathogenic strains of common plant pathogens such as *Penicillium* spp., *Colletotrichum* spp., and *Fusarium* spp. have also shown potential in controlling multiple fungal diseases. Despite being a widely investigated research area globally, the practical application of endophytic fungi in the fields remains limited. Therefore, assessing the efficacy in field applications of these fungi holds a significant importance in determining their potential use as biofungicides, while searching for novel fungal strains as antagonists. Thus, endophytic fungi may hold a promising solution for agricultural disease management and crop protection by serving as effective antagonists against plant pathogenic fungi in food crops.

## Figures and Tables

**Table 1:** Examples of phytohormones synthesized by endophytic fungi in food crops.

Host Plant	Endophytic Fungus	Hormone Secreted	Function of the Hormone	References
*Cucumis sativus* (Cucumber)	*Phoma glomerate* and *Penicillium* sp.	Gibberellic acid, IAA	Enhance plant biomass and growth parameters, facilitate the assimilation of essential nutrients, and reduce Na toxicity in drought conditions	[38]
*Glycine max* (Soybean)	*Cladosporium sphaerospermum*	Gibberellic acid	Stimulate plant growth of rice and soybean	[37]
	*Porostereum spandiceum*	Gibberellins	Promote seed germination of soybean, while saving plants from normal and salt-affected conditions, increase chlorophyll content	[39]
*Helianthus annus* (Sunflower)	*Penicillium citrinum*, *Aspergillus terreus*	IAA, Gibberellic acid	Promote plant growth of sunflower and regulate hormone signaling networks	[40]
*Oryza granulata* (Wild rice)	*Falciphora oryzae*	IAA	Improve lateral root growth, while reducing the primary root length	[41]
*Oryza sativa* (Rice)	*Phoma* sp. and *Penicillium* sp.	Gibberellins, IAA	Promote shoot and growth of rice during stress conditions such as salinity and drought	[38]
*Trapa japonica* (Water chestnut)	*Galactomyces geotrichum*	Jasmonic acid	Induce systemic resistance in soybean	
*Withenia somnifera* (Ashwagandha/Winter cherry)	*Aspergillus awamori*	Indole-3-acetic acid (IAA)	Stimulate plant growth of maize	[42]
*Zea mays* (Maize)	*Trichoderma atrovorode*	Salicylic acid, Absisic acid, Jasmonic acid	Control pathogenicity of *Fusarium verticillioides* in maize	[43]

**Table 2:** Endophytic fungal secondary metabolites and their bioactivities.

Host Plant	Endophytic Fungus	Secondary Metabolite	Bioactivity	References
*Camellia sinensis* Theaceae (Tea)	*Pestalotiopsis theae*	Punctaporonin H	Cytotoxicity and antibacterial activity	[34]
*Chaetomium seminudum* (Brown rice)	*Chaetomium seminudum*	Chaetosemins B	Antifungal activity against *Gibberella saubinetti* and *Magnaporthe oryzae* (*Pyricularia oryzae*)	
	*Chaetomium seminudum*	Chaetosemins C	Antioxidant activity	
*Garcinia adulcis* (Yellow mangosteen/Mundu)	*Phomopsis* spp.	Phomoenamide, Phomonitroester	Antitubercular activity against *Mycobacterium tuberculosis*	[37]
*Ginkgo biloba* (Gingko/Maidenhair)	*Fusarium oxysporum*	Ginkolide B	Antiallergic and anti-inflammatory properties	[22]
*Mangifera casturi* Kosterm (Kasturi mango)	*Aspergillus oryzae*	Kojic acid	Antioxidant activity	[44]
	*Aspergillus minisclerotigens*	Dihydropyran	Antioxidant activity	
*Oryza sativa* (Rice)	*Biscogniauxia cylindrospora*	Isofraxidin	Antibacterial, anticancer, and antioxidant activities	[22]
	*Annulohypoxylon boveri var. microspora*	Cinnamic acid	Antibacterial and antioxidant activities	
*Pandanus amaryllifolius* (Pandan)	*Diaporthe* sp.	Benzopyran, Diaportheone A and B	Antitubercular activity against *Mycobacterium tuberculosis*	[37]
*Piper nigrum* (Black pepper)	*Colletotrichum gloeosporioides*	Piperine	Antioxidant, antidiabetic, antibacterial, and antidiarrheal activities	[22]
*Triticum aestivum* (Wheat)	*Nigrospora oryzae*	Pipecolisporin	Antiparasitic activity against *Trypanosoma cruzi* and *Plasmodium falciparum*	[45]
*Vanilla albindia* (Vanilla)	*Phomopsis archeri*	Phomoxanthones A–C	Antimalarial activity	[37]
*Zea mays* (Maize)	*Acremonium zeae*	Pyrrocidine A and B	Antibacterial activity	[46]
*Zea mays* (Maize)	*Fusarium* sp.	Caffeine	Antifungal activity against *Alternaria alternata*	[45]

## Data Availability

No new data were created or analyzed in this study.

## Supplementary Materials

The following supporting information can be downloaded at: https://www.mdpi.com/article/10.3390/jof10090606/s1, Table S1: Summary of the antagonistic mechanisms of various endophytic fungi against different plant pathogenic fungi.
