# Supplementary material for: Exploring Endophytic Fungi as Natural Antagonists against Fungal Pathogens of Food Crops
Source: J Fungi (Basel). 2024 Aug 26;10(9):606. doi: 10.3390/jof10090606 (PMC11433156; doi:10.3390/jof10090606)
Supplement: Supplementary file 1 [file jof-10-00606-s001.zip › jof-3135172-supplementary.pdf]

Supplementary Materials

Table S1: Antagonistic activity of different endophytic fungi against phytopathogens in food crops with their antagonistic mechanisms.

Crop	Endophytic fungi	Pathogenic fungi	Fungal disease	Antagonistic mechanisms/ or bioactive agents	References
**Avocado (*Persea americana*)**	*Trichoderma harzianum*	*Colletotrichum gloeosporioides*, *Diaporthe* sp., *Neofusicoccum parvum* and *Phomopsis perseae*	Soft rot, Anthracnose, Stem end rot, Fruit rot, Phomopsis spot	Hyperparasitism, Inhibition halo formation	Lopez-Lopez *et al*. (2022)
**Cucumber (*Cucumis sativus*)**	*Trichoderma atroviride*	*Fusarium oxysporum*	Root rot disease	Inhibit the mycelial growth of pathogen by generating volatile & non-volatile metabolites	Zhang *et al*. (2022)
**Soybean (*Glycine max*)**	*Trichoderma* sp.	*Fusarium oxysporum*	Root rot disease	Mycelial inhibition by coiling around and arriving at the hyphae, Dissolve the cell walls of the target fungal species	Xu *et al*. (2022)
**Wheat (*Triticum aestivum*)**	*Trichoderma harzianum* and *T. viride*	*Alternaria* spp., *Bipolaris* spp., *Drechslera* spp., *Fusarium* spp.	Black point disease	Produce antibiotics (Cyclooctanol: *T. viride*, 6-pentyl-pyrone: *T. harzianum*)	Yassin *et al*. (2022)
**Maize (*Zea mays*)**	*Trichoderma harzianum* and *T. viride*	*Fusarium verticillioides, F. proliferatum*	Stalk rot infection	Inhibit mycelial growth, produce antibiotics of acetonic extract which contains palmitic acid, acetic acid, oleic acid, propyl benzene like bioactive metabolites	Yassin *et al*. (2021)
**Maize (*Zea mays*)**	*Trichoderma asperellum*	*Magnaporthiopsis maydis*	Late wilt disease	Produce 6-Pentyl-α-pyrone antifungal compound	Degani *et al*. (2021)
**Cucumber (*Cucumis sativus*)**	*Trichoderma atroviride*	*Rhizoctonia solani*		Formation of signaling molecules such as hydrogen peroxide (H2O2), nitric oxide (NO), salicylic acid (SA), and derivatives of it	Nawrocka *et al*. (2019)

				such as methyl salicylate (MeSA) and octyl salicylate (OSA)	
**Chili pepper (*Capsicum annuum*)**	*Trichoderma koningiopsis*	*Colletotrichum gloeosporioides*	Anthracnose	Produce antifungal metabolites such as ethyl hexadecanoate, azetidine, and 2-phenylethanol by suppressing fungal mycelial growth, Distorting & lysis the shape of hyphae, produce cell wall degrading enzymes, Reduce lesion size	Ruangwong *et al*. (2021)
**Peanut (*Arachis hypogaea*)**	*Trichoderma koningiopsis*	*Macrophomina phaseolina*	Charcoal rot	Inhibit the radial growth of pathogen	Martinez-Salgado *et al*. (2021)
**Hot pepper (*Capsicum annuum*)**	*Trichoderma harzianum* and *T. viride*	*Fusarium oxysporum* f. sp. *capsici*	Fusarium wilt	Produce hydrolytic enzymes that have the mycoparasitism ability	Girma (2022)
**Banana (*Musa acuminata*)**	*Trichoderma reesei*	*Fusarium oxysporum* f. sp. *cubense*	Fusarium wilt	Produce antifungal compounds such as iturin C19, ß-caryophyllene, soyasapogenol rhamnosyl glucuronide, catechin-o-gallate, peptaibols, fenigycin, gallocatechin-o-gallate, and anthocyanin, Mycoparasitism, Reduce fungal toxins such as beauveric acid, fusarin C, and fusaristatin A	Damodaran *et al*. (2020)
**Soybean (*Glycine max*)**	*Trichoderma* sp.	*Phakopsora pachyrhizi*	Asian soybean rust	Produce antifungal compounds such as 6-pentyl-α-pyrone and harzianolide to suppress the uredospore germination	El-Hasan *et al*. (2022)
**Tea (*Camellia sinensis*)**	*Trichoderma viride*	*Fusarium solani, Pestalotia theae*	Dieback disease, Grey blight disease	Produce extracellular enzymes such as β-1,3-glucanase, cellulase,	Naglot *et al*. (2015)

				amylase, pectinase, chitinase, and protease, Alternate hyphal morphology like swelling, distortion, & cytoplasm aggregation	
**Banana (*Musa acuminata*)**	*Penicillium citrinum*	*Fusarium oxysporum* f. sp. *cubense*	Fusarium wilt	Produce biochemicals including peroxidase, phenylalanine ammonia-lyase, and polyphenol oxidase as induced resistance response	Ting *et al.* (2012)
**Mung bean (*Vigna radiata*)**	*Penicillium crustosum, P. digitatum, P. janczewskii, P. oxalicum* and *P. verrucosum*	*Phoma herbarum*	Leaf spot disease	Disrupt MAPK cascade	Shafique *et al.* (2023)
**Cabbage (*Brassica oleracea*), Potato (*Solanum tuberosum*)**	*Penicillium* sp.	*Fusarium oxysporum*	Fusarium wilt		
**Chickpea (*Cicer arietinum*)**	*Penicillium citrinum*	*Botrytis cinera*	Botrytis gray mold	Secrete citrinin	
**Oilseed rape (*Brassica napus*)**	*Penicillium oxalicum*	*Sclerotinia sclerotiorum*	Stem rot disease	Produce antifungal substances	Yang *et al.* (2007)
**Apple (*Malus domestica*)**	*Fusarium solani*	*Valsa ceratosperma*	Japanese apple canker disease	Produce antifungal substances including cyclosporin A and C	Sawai *et al.* (1981)
**Wheat (*Triticum aestivum*)**	*Talaromyces trachyspermus*	*F. culmorum, Fusarium graminearum, F. pseudograminearum*	Crown rot disease		Zhao *et al.* (2022)
**Muskmelon (*Cucumis melo*)**	*Nigrospora sphaerica*	*Monosporascus cannonballus*	Vine decline disease	Inhibit mycelial growth by creating morphological abnormalities like shrinkage, turgidity, & disintegration	Al-Badi *et al.* (2020)
**Almond (*Prunus dulcis*), Citrus (*Citrus limon*), Peach (*Prunus persica*), Olive (*Olea europaea*),**	*Alternaria* sp., *Arthrinium* sp., *Aspergillus niger, Epicoccum* sp., *Fusarium* sp. and *Nigrospora oryzae*	*Colletotrichum acutatum Simmond*	Blight, Anthracnose	Produce volatile inhibitory metabolites including 4-methyl quinazoline, benzyl alcohol, phenyl ethyl	Landum *et al.* (2016)

**Strawberry (*Fragaria×ananassa*)**				alcohol, benzothiazole, lilial, and galaxolide	
**Potato (*Solanum tuberosum*)**	*Acremonium* sp., *Gliocladium viride*, *Paecilomyces marquandii*, *P. sulphurellus*, *Penicillium camemberti*, *P. expansum*, *P. frequentans*, *P. nigricans*, *P. olsonii*, *P. phialosporum*, *Sporothrix schenckii*, *Sporothrix* sp. and *Verticillium dahliae*	*Rhizoctonia solani*	Black scurf disease, Stem canker disease	Release fungistatic substances, Create coiling of hyphae	Demirci *et al*. (2011)
**Guava (*Psidium guajava*)**	*Pichia anomala*	*Botryodiplodia theobromae*	Diplodia rot disease	Produce pectinase and cellulase enzymes	Hashem & Alamri (2009)
**Grapevine (*Vitis vinifera*)**	*Pythium oligandrum*	*Botrytis cinerea*	Gray mold disease	Cuticle thickness modification, Phenolic compound accumulation, Apposition of the cell wall	Mohamed *et al*. (2007)
**Rice (*Oryza sativa*)**	*Cladosporium cladosporioides*		Rice blast disease	Produce lytic enzymes, Hinder mycelial growth, conidial germination, & appressorium formation	Chaibub *et al*. (2020)
**Onion (*Allium cepa*)**	*Epicoccum nigrum*, *Penicillium oxalicum*, *Trichoderma harzianum*	*Alternaria porri*	Purple blotch disease	Trichoderma show mycoparasitism & competition, E. nigrum produce bioactive compounds such as epirodins, avipin, and epicorazines, P. oxalicum secrete extracellular lytic enzymes such as chitinases, α-1,3-glucanases, and cellulases	Abdel-Hafez *et al*. (2015)
